# Precise fabrication of single-atom alloy co-catalyst with optimal charge state for enhanced photocatalysis

**DOI:** 10.1093/nsr/nwaa224

**Published:** 2020-09-03

**Authors:** Yating Pan, Yunyang Qian, Xusheng Zheng, Sheng-Qi Chu, Yijun Yang, Chunmei Ding, Xi Wang, Shu-Hong Yu, Hai-Long Jiang

**Affiliations:** Hefei National Laboratory for Physical Sciences at the Microscale, CAS Key Laboratory of Soft Matter Chemistry, Department of Chemistry, University of Science and Technology of China, Hefei 230026, China; Hefei National Laboratory for Physical Sciences at the Microscale, CAS Key Laboratory of Soft Matter Chemistry, Department of Chemistry, University of Science and Technology of China, Hefei 230026, China; National Synchrotron Radiation Laboratory (NSRL), University of Science and Technology of China, Hefei 230029, China; Beijing Synchrotron Radiation Facility, Institute of High Energy Physics, Chinese Academy of Sciences, Beijing 100049, China; Key Laboratory of Luminescence and Optical Information, Ministry of Education, Department of Physics, School of Science, Beijing Jiaotong University, Beijing 100044, China; Dalian National Laboratory for Clean Energy, State Key Laboratory of Catalysis, Dalian Institute of Chemical Physics, Chinese Academy of Sciences, Dalian 116023, China; Key Laboratory of Luminescence and Optical Information, Ministry of Education, Department of Physics, School of Science, Beijing Jiaotong University, Beijing 100044, China; Hefei National Laboratory for Physical Sciences at the Microscale, CAS Key Laboratory of Soft Matter Chemistry, Department of Chemistry, University of Science and Technology of China, Hefei 230026, China; Hefei National Laboratory for Physical Sciences at the Microscale, CAS Key Laboratory of Soft Matter Chemistry, Department of Chemistry, University of Science and Technology of China, Hefei 230026, China

**Keywords:** metal-organic framework (MOF), single-atom alloy (SAA), co-catalyst, surface charge state, photocatalysis

## Abstract

While the surface charge state of co-catalysts plays a critical role for boosting photocatalysis, studies on surface charge regulation via their precise structure control remain extremely rare. Herein, metal-organic framework (MOF) stabilized bimetallic Pd@Pt nanoparticles, which feature adjustable Pt coordination environment and a controlled structure from core-shell to single-atom alloy (SAA), have been fabricated. Significantly, apart from the formation of a Mott-Schottky junction in a conventional way, we elucidate that Pt surface charge regulation can be alternatively achieved by changing its coordination environment and the structure of the Pd@Pt co-catalyst, where the charge between Pd and Pt is redistributed. As a result, the optimized Pd_10_@Pt_1_/MOF composite, which involves an unprecedented SAA co-catalyst, exhibits exceptionally high photocatalytic hydrogen production activity, far surpassing its corresponding counterparts.

## Teaser

The MOF-stabilized Pd_10_@Pt_1_ single-atom alloy was fabricated, featuring optimized Pt surface charge state and greatly boosted photocatalysis, thanks to the charge redistribution regulated by Pt coordination environment and Pd@Pt microstructure.

## INTRODUCTION

Photocatalysis, converting solar to chemical energy, represents a very promising solution to current energy and environmental issues [1–4]. Photocatalytic performance depends largely on the surface charge state of a catalyst, as it directly correlates with charge transfer from the catalyst surface to reactant molecules as well as adsorption and activation of the latter [5–7]. Various strategies have been developed to control the surface charge states of photocatalytic materials [6,7]. Amongst them, the construction of a Schottky junction by introducing co-catalyst is one of the most common and effective strategies via increasing the active sites, promoting charge separation, accelerating charge transfer and minimizing reaction over-potentials [8–12]. Recent studies on co-catalysts are mostly related to the development of non-noble metal co-catalysts, the control of particle sizes, particle distribution, exposed crystal facets as well as their interface contact with photocatalysts, and even synergistic effect among various co-catalysts [6,8–12]. Nonetheless, specialized regulation on the surface charge state of co-catalysts, especially by changing their microstructure, remains largely unexplored, though this would be highly desired to boost the photocatalysis [6,10,12].

To achieve surface charge regulation, the introduction of bimetallic nanoparticles (NPs) as co-catalysts, in which one metal behaves as the active site and the other as charge regulator, would be a promising solution. Of the common alloyed or core-shell bimetallic NPs, the core-shell architecture featuring active metal shell would be preferred, for ease of access to active sites. The gradually decreased content of active metal would cause its varying coordination environment and surface charge. Particularly, atomically dispersed active metal atoms will be formed when their content goes down to a certain amount. The microstructure of active metal in its atomically dispersed form on the other metal support is called single-atom alloy (SAA) [13–17]. Compared with other single-atom catalysts (SACs) [18,19], SAA catalysts present geometric/strain, electronic and synergistic effects originating from bimetallic components [13–17,20,21], causing the *d*-band center change of the surface active metals and thereby affecting their catalytic performance [13–17]. Surprisingly, the related exploration on SAA catalysts toward their photocatalysis has not yet been reported. In this context, it is imperative to investigate the structural evolution of bimetallic NPs from core-shell to SAA, which might be able to systematically regulate the charge state of surface active metal atoms, for boosting photocatalysis.

Meanwhile, the ideal photosensitizers, which not only have intimate contact with but are also able to stabilize SAA co-catalysts, are highly desired. In this regard, metal-organic frameworks (MOFs) [22–27], a class of crystalline porous materials featuring well-defined and tailored structures, high surface area, as well as semiconductor-like behavior [28–31], would be ideal candidates. Given the well-tuned conjugation of linkers, MOFs are able to harvest solar light in a broad spectral range and have shown their great potential as photosensitizers and photocatalysts [32–45]. Various co-catalysts, particularly the most classical Pt NPs with low over-potential, have been introduced and well stabilized by MOFs, greatly accelerating the charge separation and photocatalysis, mainly via the formation of a Pt-MOF ‘Schottky junction’ [9–11,46–49]. Therefore, MOFs would be very promising in stabilizing single atomic Pt-based bimetallic NPs for photocatalysis.

**Figure 1.**
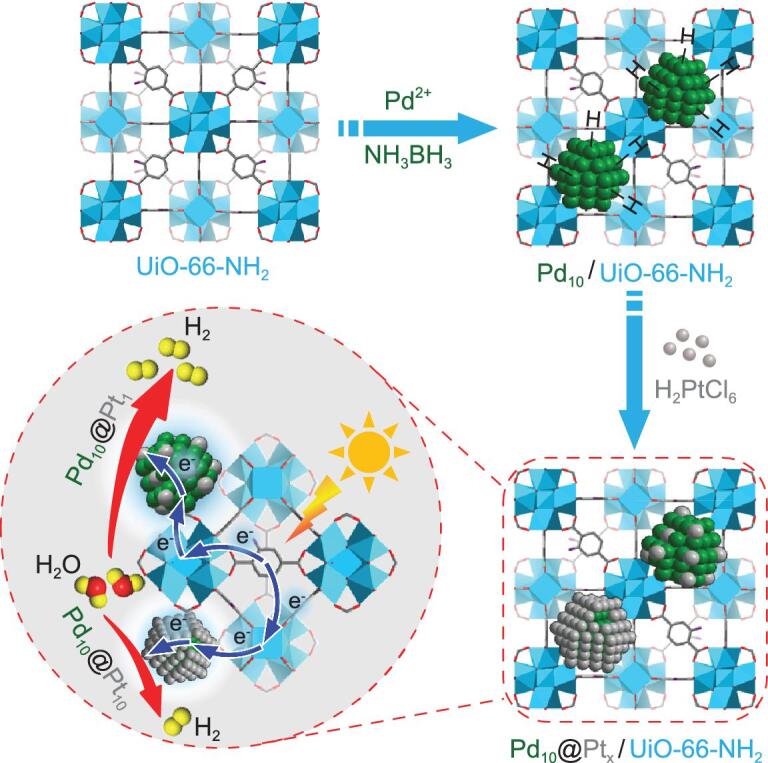
Schematic illustration. Illustration showing the synthetic strategy for Pd_10_@Pt_x_/UiO-66-NH_2_ toward photocatalytic hydrogen production, highlighting the much higher activity of Pd_10_@Pt_1_ SAA than Pd_10_@Pt_10_ core-shell NPs.

Bearing the above in mind, bimetallic core-shell Pd@Pt NPs have been *in situ* fabricated and stabilized by a representative robust MOF with good light harvesting capability, UiO-66-NH_2_, to afford Pd_10_@Pt_x_/UiO-66-NH_2_ composite (the 10 and x mean the mass permillage, at wt‰, of Pd and Pt to the MOF in synthesis, respectively). Remarkably, the Pt component on the Pd surface can be precisely controlled from the shell-form to single-atom dispersion. Along with structural evolution of Pd@Pt NPs from core-shell to SAA, the Pt coordination environment changes and the regulation of its surface charge state is accordingly achieved. The obtained Pd_10_@Pt_x_/UiO-66-NH_2_ exhibits excellent photocatalytic activity toward hydrogen production by water splitting (Fig. 1). Particularly, thanks to the charge redistribution, the optimized Pd_10_@Pt_1_/UiO-66-NH_2_ with SAA structure possesses superb activity, far surpassing the monometallic NPs and their physical mixture stabilized by the MOF. Though both size and location of Pt NPs have been reported to be crucial for charge separation efficiency and activity [9,11], to the best of our knowledge, there has not yet been explored on regulating the Pt coordination environment and its surface charge state by controlling co-catalyst structure in the photocatalytic system. Moreover, this is the first work investigating the SAA catalyst for photocatalysis.

## RESULTS

### The fabrication and characterizations of Pd_10_@Pt*_x_*/UiO-66-NH_2_

The Pd precursor was impregnated to UiO-66-NH_2_ and subsequently reduced by ammonia borane (NH_3_BH_3_) to give Pd_10_/UiO-66-NH_2_. Upon the exhaustion of NH_3_BH_3_, different amounts of Pt precursor are added into the synthetic system. The surface Pd-H species, generated during NH_3_BH_3_ hydrolysis, would serve as both nucleation seed and reducing agent for the Pt precursor, generating core-shell-structured Pd_10_@Pt_x_ (x = 0.3, 1, 5, 10) NPs stabilized by UiO-66-NH_2_ (Fig. 1) [50–55].

**Figure 2.**
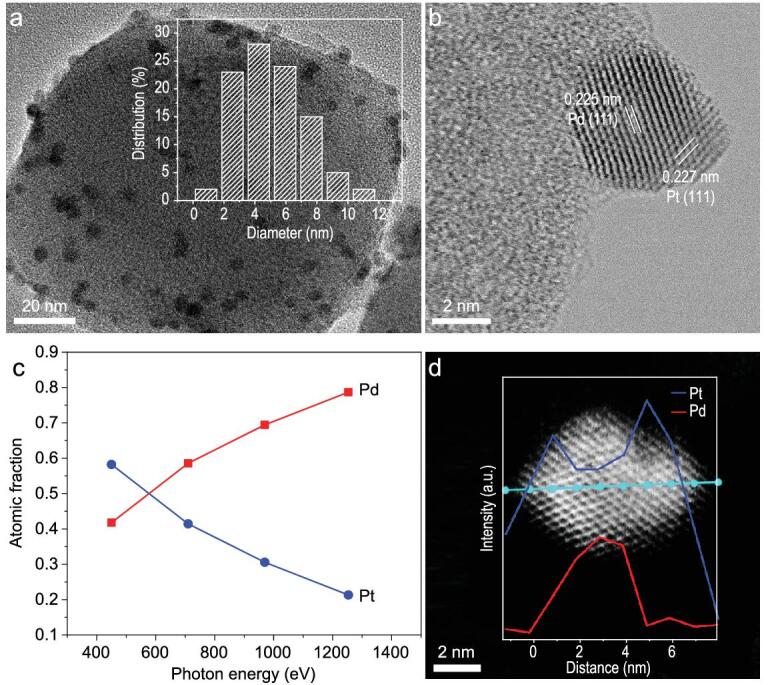
Structural characterizations of Pd_10_@Pt_10_ core-shell NPs. (a) TEM (inset: size distribution for Pd_10_@Pt_10_ NPs) and (b) HRTEM images of Pd_10_@Pt_10_/UiO-66-NH_2_. (c) Dependence of Pd and Pt atomic fractions for the Pd_10_@Pt_10_/UiO-66-NH_2_ (treated with H_3_PO_4_) as a function of photon energy. (d) Elemental line-scanning spectra across a single Pd_10_@Pt_10_ particle (stabilized by UiO-66-NH_2_) along the direction marked by a cyan line.

Powder X-ray diffraction (PXRD) patterns indicate that the high crystallinity of UiO-66-NH_2_ is well maintained during the introduction of Pd@Pt NPs (Supplementary Fig. 1). The actual contents of Pd and Pt are determined by the inductively coupled plasma atomic emission spectrometer (ICP-AES) (Supplementary Table 1). All Pd_10_@Pt_x_/UiO-66-NH_2_ have similar Pd loadings, while the Pt steadily increases as more Pt precursors are introduced. Transmission electron microscopy (TEM) observation shows that Pd NPs in Pd_10_/UiO-66-NH_2_ are well dispersed with average sizes of 4.01 nm (Supplementary Fig. 2). Upon Pt deposition, the Pd@Pt sizes slightly increase with higher Pt contents (Fig. 2a and b, Supplementary Figs 3–5 and Supplementary Table 2), in good agreement with the expectation of the epitaxial Pt shell growth on Pd NPs. Meanwhile, scanning electron microscope (SEM) images of Pd_10_@Pt_x_/UiO-66-NH_2_ indicate the maintained morphology of UiO-66-NH_2_ after introducing Pd@Pt NPs (Supplementary Figs 6 and 7).

To verify whether the Pd@Pt core-shell structure has been successfully constructed, multiple characterizations have been adopted by taking Pd_10_@Pt_10_/UiO-66-NH_2_ as a representative. A high-resolution TEM (HRTEM) image reveals the Pd core is covered by a Pt shell, as evidenced by their exposed {111} facets with clear lattice fringes (Fig. 2b). The spatial distribution of the constituent elements of Pd@Pt NPs is detected by synchrotron radiation photoemission spectroscopy (SRPES) spectra for Pd 3*d* and Pt 4*f*, at photon energies of 450, 710, 970 and 1253.6 eV, respectively (Supplementary Figs 8–10) [56,57]. The peaks at 340.3 and 335.0 eV can be assigned to Pd 3*d*_3/2_ and Pd 3*d*_5/2_ of metallic Pd^0^, respectively [56], whereas the peaks at 74.3 and 70.9 eV clearly correspond to Pt^0^ (Supplementary Fig. 10) [58,59]. The outside-in atomic fractions of Pd and Pt in Pd@Pt NPs can be obtained from their peak areas calibrated by the beam flux and photoionization cross-section at the corresponding photon energy. As a direct result, a decreased atomic fraction of Pt and an increased atomic fraction of Pd unambiguously suggest that Pt stays on the external surface of Pd, pointing to a Pd_10_@Pt_10_ core-shell structure (Fig. 2c). More intuitively, the core-shell structure can also be clearly identified by elemental line-scanning spectra for a single Pd_10_@Pt_10_ particle (Fig. 2d and Supplementary Fig. 11).

**Figure 3.**
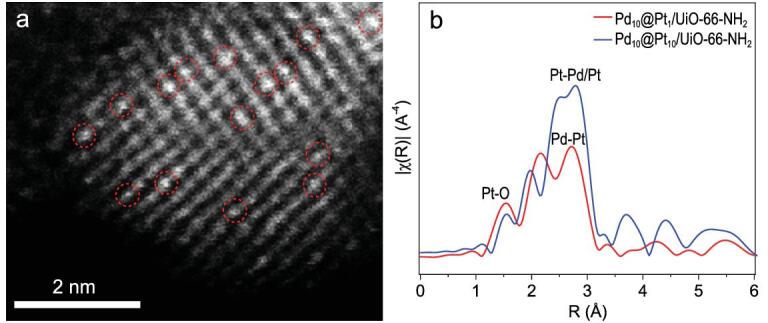
Structural characterizations of Pd_10_@Pt_1_ SAA. (a) HAADF-STEM image of Pd_10_@Pt_1_/UiO-66-NH_2_. The single Pt atoms are highlighted by the red circles and the lattice fringes are from Pd while the MOF is shaded in black background. (b) The Pt L_3_-edge FT-EXAFS spectra for Pd_10_@Pt_1_/UiO-66-NH_2_ and Pd_10_@Pt_10_/UiO-66-NH_2_.

Furthermore, the thickness of the Pt shell can be precisely controlled by varying the ratio of Pd/Pt precursors. As the Pt content decreases to give Pd_10_@Pt_1_/UiO-66-NH_2_, strikingly, the high-angle annular dark-field scanning transmission electron microscopy (HAADF-STEM) image indicates that Pt is atomically dispersed on the Pd surface (Fig. 3a). Fourier transformed extended X-ray absorption fine structure (FT-EXAFS) and X-ray absorption near-edge structure (XANES) analyses provide more detailed structural information on Pd@Pt NPs as well as the Pt coordination environment (Fig. 3b, Supplementary Fig. 12 and Supplementary Tables 3 and 4). The absence of Pt–Pt peak in the Pt L_3_-edge FT-EXAFS spectrum of Pd_10_@Pt_1_/UiO-66-NH_2_ further demonstrates that the Pt, in its single-atom form, locates on the Pd surface (Fig. 3b) [58,59]. The weak peak at 1.5 Å is ascribed to the Pt-O bonding, which is possibly caused by partial surface oxidation. The slightly oxidized Pt also gives rise to a minor increase in white line intensity in the Pt L_3_-edge XANES spectrum for Pd_10_@Pt_1_/UiO-66-NH_2_ (Supplementary Fig. 13) [58,59]. All the HAADF-STEM, FT-EXAFS and XANES results unambiguously confirm that the SAA structure is formed in Pd_10_@Pt_1_/UiO-66-NH_2_. In sharp contrast to Pd_10_@Pt_1_/UiO-66-NH_2_, the Pd_10_@Pt_10_/UiO-66-NH_2_ apparently shows the presence of a Pt-Pd/Pt coordination bond. Moreover, the Pt-Pd coordination number in Pd_10_@Pt_10_/UiO-66-NH_2_ is much lower than that in Pd_10_@Pt_1_/UiO-66-NH_2_ (Supplementary Tables 3 and 4), indicating that Pt possibly presents a cumulative atomic layer-like distribution on the Pd surface in the former sample. Therefore, the Pd@Pt NPs are successfully stabilized by UiO-66-NH_2_, and the coordination environment of Pt is adjustable along with precise regulation of Pt shells to single Pt atoms on the surface of Pd NPs.

### Charge redistribution effect

**Figure 4.**
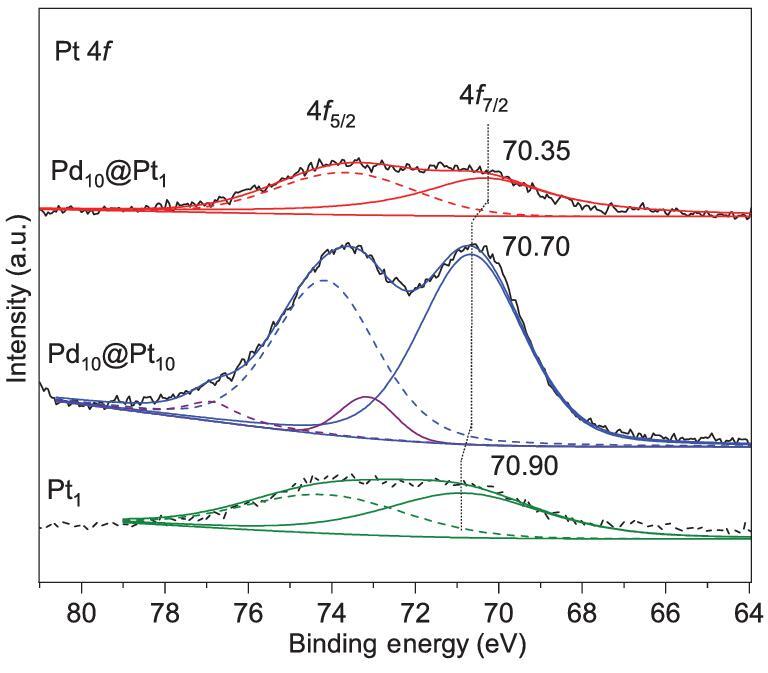
XPS results. The Pt 4*f* XPS spectra for Pt_1_/UiO-66-NH_2_, Pd_10_@Pt_1_/UiO-66-NH_2_ and Pd_10_@Pt_10_/UiO-66-NH_2_ (the purple solid and dashed lines represent slightly oxidized Pt species).

Given the different working functions of Pd and Pt (Supplementary Fig. 14), to balance the Fermi distribution of electrons in Pd@Pt NPs, electrons tend to leave Pd (the lower work function and electrons being less tightly bound) and travel to Pt (the higher work function) at their interface, causing an altered surface charge state of Pt. It is assumed that, as the structure of Pd@Pt NPs evolves from core-shell to SAA, charge redistribution (charge density change between the core and the surface metals) would take place and this affects the Pt surface charge state [12,60]. The work function calculations for Pd@Pt with SAA and core-shell structure support the above point (Supplementary Fig. 14). To further verify this hypothesis, X-ray photoelectron spectroscopy (XPS) measurements have been conducted (Fig. 4). Compared to the Pt^0^ 4*f*_7/2_ peak at 70.9 eV for Pt_1_/UiO-66-NH_2_, Pd_10_@Pt_1_/UiO-66-NH_2_ presents lower binding energy to varying degrees, reflecting that the Pt surface in Pd@Pt NPs becomes electron-rich and charge redistribution effect between Pd and Pt exists. Moreover, when less Pt_x_ (x value from 10 to 1) is covered, the Pt coordination number increases via surrounding Pd-Pt bonds (Supplementary Tables 2–4) and the Pt binding energy decreases (Fig. 4), further manifesting gradually enhanced charge redistribution effect and the most electron-rich Pt in Pd_10_@Pt_1_ NPs. In fact, from the aforementioned SRPES spectra of Pt 4*f* (Supplementary Fig. 10), when the probe depth gets close to the Pd core as photon energy increases, the Pt binding energy appears as a negative shift, which further supports the above conclusion.

**Figure 5.**
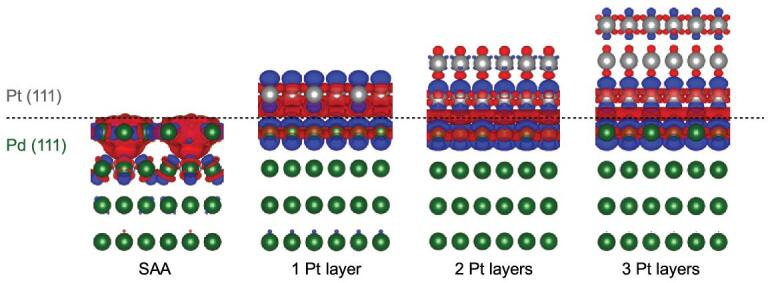
Charge redistribution indicated by differential charge density. Differential charge density of Pd@Pt NPs with SAA and core-shell structure featuring different Pt layer thicknesses. The red and blue colors represent the increase and decrease in electron density, respectively.

In addition, the differential charge density of Pd@Pt NPs (from core-shell structure with different Pt thicknesses to SAA) based on density-functional theory (DFT) calculations affords consistent results, manifesting the enhanced charge accumulation on the Pt surface along with the decrease of Pt layer numbers (i.e. from core-shell to SAA structure, finally) (Fig. 5, red color). From the differential charge density, the Bader charge of each Pt atom and the binding energy shift of Pt 4*f*_7/2_ show similar slopes along with the increased Pt layer thickness, indicating the consistent charge redistribution trend in simulation and experiment (Supplementary Fig. 15, Supplementary Tables 5 and 6).

### Photocatalysis and photoelectrochemical measurements

**Figure 6.**
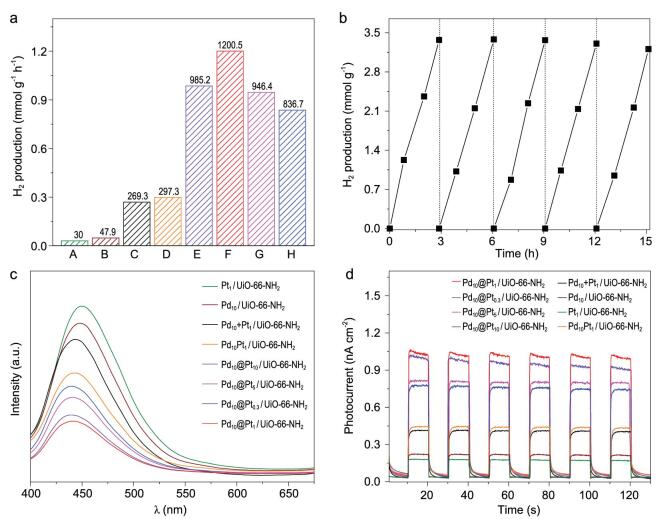
Photocatalytic results and photoelectrochemical measurements. (a) Photocatalytic hydrogen production rate of: A, Pt_1_/UiO-66-NH_2_; B, Pd_10_/UiO-66-NH_2_; C, Pd_10 _+ Pt_1_/UiO-66-NH_2_; D, Pd_10_Pt_1_/UiO-66-NH_2_ and E–H, Pd_10_@Pt_x_/UiO-66-NH_2_ (x = E, 0.3; F, 1; G, 5; H, 10). (b) Recycling performance of Pd_10_@Pt_1_/UiO-66-NH_2_. (c) Photoluminescence spectra under excitation at λ = 360 nm. (d) Photocurrent response.

Encouraged by the electron-rich Pt surface in Pd_10_@Pt_x_/UiO-66-NH_2_, we set out to investigate the influence of charge redistribution for photocatalytic H_2_ production (Fig. 6a). Prior to the measurement, UV-Vis absorption spectra of Pd_10_@Pt_x_/UiO-66-NH_2_ have been examined to demonstrate their similar light absorption (Supplementary Fig. 16). As expected, as the Pt loading in Pd@Pt NPs decreases (x from 10 to 1), the Pt-Pd coordination number increases, charge redistribution effect strengthens and Pt becomes more electron-rich. Accordingly, the activity of Pd_10_@Pt_x_/UiO-66-NH_2_ exhibits gradual increase. Strikingly, Pd_10_@Pt_1_/UiO-66-NH_2_ featuring SAA structure achieves the highest photocatalytic H_2_ production rate, 1200.5 μmol · g^−1^ · h^−1^, among all investigated samples, which is 25, 30 and ∼4.5 times higher than that of Pd_10_/UiO-66-NH_2_, Pt_1_/UiO-66-NH_2_, Pd_10 _+ Pt_1_/UiO-66-NH_2_ with a mixture of monometallic NPs, and Pd_10_Pt_1_/UiO-66-NH_2_, respectively (Fig. 6a and Supplementary Figs 17–20). When the Pt content continues to decline (x from 1 to 0.3), unexpectedly, the photocatalytic hydrogen production activity slightly decreases, possibly due to the decreased Pt sites in the catalyst.

The calculated Gibbs free energy (ΔG_H_*) of Pt and Pd atoms in the Pd_10_@Pt_1_ SAA structure indicates that Pt is the more favorable active site than Pd for hydrogen production (Supplementary Fig. 21). Together with the overwhelmingly higher activity of Pd_10_@Pt_1_/UiO-66-NH_2_ than Pd_10_/UiO-66-NH_2_, the H_2_ generation taking place on Pd should be negligible and turnover frequencies of Pt (TOF_Pt_) are a reasonable descriptor for the activity for the catalysts in SAA structure. The significantly higher TOF_Pt_ for Pd_10_@Pt_0.3_/UiO-66-NH_2_ and Pd_10_@Pt_1_/UiO-66-NH_2_ than all other counterparts further indicates the superiority of SAA structure in photocatalysis (Supplementary Table 7).

In addition, this optimized Pd_10_@Pt_1_/UiO-66-NH_2_ catalyst shows good recyclability and its activity does not present obvious decay in five consecutive runs and a continued test for 25 h (Fig. 6b, Supplementary Fig. 25). Thanks to the good stability, PXRD patterns confirm that the structural integrity and crystallinity of the MOF in all samples are well retained after catalysis (Supplementary Fig. 22). Especially, both the SAA structure and the dispersion of Pd_10_@Pt_1_ NPs are almost maintained, thanks to the excellent stabilization effect by the MOF (Supplementary Figs 23 and 24).

The activity promotion by surface charge regulation based on Pd_10_@Pt_x_/UiO-66-NH_2_ for photocatalysis has also been demonstrated by enhanced charge transfer. Photoluminescence (PL) emission spectra for all samples show that Pd_10_@Pt_1_/UiO-66-NH_2_ has the weakest fluorescence intensity, indicating its best charge separation efficiency, and the peak intensity order is in perfect reverse correlation with that of photocatalytic activity (Fig. 6c). The photocurrent and electrochemical impedance spectroscopy (EIS) measurements also manifest such a trend (Fig. 6d and Supplementary Fig. 26), further confirming the discriminative charge separation efficiency and being in good agreement with the results by XPS, SRPES spectra and DFT calculations, and well explaining the activity trend. Moreover, the cathodic polarization curves indicate Pd_10_@Pt_1_/UiO-66-NH_2_ has lower over-potential for hydrogen evolution (Supplementary Fig. 27). Therefore, the regulation of Pt surface charge state by charge redistribution at the interface of Pd and Pt plays an important role in optimizing photocatalytic performance. Base on the results above and previous reports [9,48,49], the photocatalytic process of Pd@Pt/UiO-66-NH_2_ is clear that the MOF is excited by visible light to produce photogenerated electron-hole pairs. Due to the Schottky junction, the electrons transfer to Pd, and finally to Pt (catalytic center), promoted by the charge redistribution effect between Pd and Pt (Fig. 1). The charge redistribution effect is influenced by the Pt coordination environment and microstructure, which causes distinct charge accumulation on the Pt surface in Pd_10_@Pt_x_/UiO-66-NH_2_, resulting in different H_2_ production activity. The results unambiguously manifest that, in addition to the well-established Schottky junction, the microstructure regulation of a co-catalyst reported herein might be another effective and novel strategy to enhance charge separation and thus photocatalysis.

## DISCUSSION

In summary, a bimetallic NPs/MOF system, Pd@Pt/UiO-66-NH_2_, has been rationally fabricated to investigate the structural influence of a Pd@Pt co-catalyst on its surface charge state and resulting photocatalytic activity. Remarkably, the Pt shell on Pd surface can be precisely controlled and the structure evolves from core-shell to SAA, so as to change the Pt coordination environment. Consequently, the surface charge on Pd and Pt is redistributed, which regulates the electronic state of Pt active sites and is found to be crucial for the resulting activity. As a result, the photocatalytic hydrogen production activity of Pd_10_@Pt_x_/UiO-66-NH_2_ can be optimized to exponentially increase by precisely fabricating the Pd_10_@Pt_1_ SAA co-catalyst, based on the rational control of Pt loading. This work not only presents SAA behaving as a very promising co-catalyst for photocatalysis for the first time, but also opens a novel avenue to the surface charge regulation of co-catalysts for boosting photocatalysis.

## METHODS

### Preparation of Pd_10_@Pt_x_/UiO-66-NH_2_

Typically, 100 mg UiO-66-NH_2_ was put in a porcelain crucible and a certain amount of 50 mg/mL Pd(NO_3_)_2_ aqueous solution (Pd/UiO-66-NH_2_ = 1 wt%) was added. Then, 200 μL MeOH was added into the mixture and rapidly stirred with a glass rod and heated at 80°C to evaporate the solution. This procedure was repeated twice and the obtained solid was dried in a 85°C drying oven for 15 min to give Pd^2+^/UiO-66-NH_2_. After that, a 15 mg sample of Pd^2+^/UiO-66-NH_2_ was dispersed in 5 mL MeOH by ultrasonication, and 5 mg NH_3_BH_3_ was added during stirring. After H_2_ bubbling was completed, a certain amount of H_2_PtCl_6_ aqueous solution (the mass ratio of Pt/Pd = 0.3, 1, 5, 10/10, respectively) was quickly added and stirred for 1 h. The solid was collected by centrifugation and washed by MeOH several times, and dried at 60°C under vacuum overnight. The product was named Pd_10_@Pt_x_/UiO-66-NH_2_ (the 10 and x mean the mass permillage, at wt‰, of Pd and Pt to the MOF in the synthesis, respectively).

### Synchrotron radiation photoemission spectroscopy (SRPES)

The SRPES spectra were measured with photon energies of 450 eV, 710 eV, 970 eV and 1253.6 eV at the BL10B beamline in the National Synchrotron Radiation Laboratory (NSRL, Hefei, China) [56,57]. Before the SRPES test, 30 mg Pd_10_@Pt_10_/UiO-66-NH_2_ was dispersed in 1 mL H_3_PO_4_ and stirred for 2 h at room temperature to dissolve the MOF to improve the electrical conductivity of the sample.

### The X-ray absorption spectra

The Pt L_3_-edge X-ray absorption spectra (XAS) data were collected at 1W1B beam line of the Beijing Synchrotron Radiation Facility (BSRF, Beijing) operated at 2.5 GeV at room temperature. The Si (111) double crystal monochromator was calibrated via Pt foil and then the Pt foil XAS data was collected as the reference spectrum. The XAS measurement for the samples was performed in the fluorescence mode using a Lytle detector. Data analysis was performed with Athena and Artemis included in the IFEFFIT version 0.8.012. A first-shell single scattering path was used in fitting the FT-EXAFS data.

### Photocatalytic experiments

Typically, 3 mg photocatalyst was dispersed in 27 mL acetonitrile containing 0.2 mL deionized water and 3 mL triethylamine (as a sacrificial reagent). Then the suspension was transferred into an optical reaction vessel (160 mL) and purged with nitrogen for 15 min to remove air. The reaction solution was irradiated by the 300 W Xe lamp (LX-300F, Japan) equipped with a UV cut-off filter (>380 nm). Hydrogen gas was measured by gas chromatography (Shimadzu GC-2014) using a thermal conductivity detector.

## Supplementary Material

nwaa224_Supplemental_Files

## References

[1] Li XB , Tung CH, Wu LZ. Semiconducting quantum dots for artificial photosynthesis. Nat Rev Chem 2018; 21: 60–173. 10.1038/s41570-018-0024-8

[2] Wang Z , Li C, Domen K. Recent developments in heterogeneous photocatalysts for solar-driven overall water splitting. Chem Soc Rev 2019; 48: 2109–25. 10.1039/C8CS00542G 30328438

[3] Schultz DM , Yoon TP. Solar synthesis: prospects in visible light photocatalysis. Science 2014; 343: 1239176. 10.1126/science.1239176 24578578 PMC4547527

[4] Wang F , Li Q, Xu D. Recent progress in semiconductor-based nanocomposite photocatalysts for solar-to-chemical energy conversion. Adv Energy Mater 2017; 7: 1700529. 10.1002/aenm.201700529

[5] Pacchioni G , Freund HJ. Controlling the charge state of supported nanoparticles in catalysis: lessons from model systems. Chem Soc Rev 2018; 47: 8474–502. 10.1039/C8CS00152A 29697127

[6] Liu L , Corma A. Metal catalysts for heterogeneous catalysis: from single atoms to nanoclusters and nanoparticles. Chem Rev 2018; 118: 4981–5079. 10.1021/acs.chemrev.7b00776 29658707 PMC6061779

[7] Tong H , Ouyang S, Bi Y et al. Nano-photocatalytic materials: possibilities and challenges. Adv Mater 2012; 24: 229–51. 10.1002/adma.201102752 21972044

[8] Ran J , Zhang J, Yu J et al. Earth-abundant cocatalysts for semiconductor-based photocatalytic water splitting. Chem Soc Rev 2014; 43: 7787–812. 10.1039/C3CS60425J 24429542

[9] Xiao JD , Shang Q, Xiong Y et al. Boosting photocatalytic hydrogen production of a metal-organic framework decorated with platinum nanoparticles: the platinum location matters. Angew Chem Int Ed 2016; 55: 9389–93. 10.1002/anie.201603990 27321732

[10] Li X , Yu J, Jaroniec M et al. Cocatalysts for selective photoreduction of CO_2_ into solar fuels. Chem Rev 2019; 119: 3962–4179. 10.1021/acs.chemrev.8b00400 30763077

[11] Xiao JD , Han L, Luo J et al. Integration of plasmonic effects and Schottky junctions into metal-organic framework composites: steering charge flow for enhanced visible-light photocatalysis. Angew Chem Int Ed 2018; 57: 1103–7. 10.1002/anie.201711725 29215207

[12] Chen F , Huang H, Guo L et al. The role of polarization in photocatalysis. Angew Chem Int Ed 2019; 58: 10061–73. 10.1002/anie.201901361 30794343

[13] Pei GX , Liu XY, Wang A et al. Ag alloyed Pd single-atom catalysts for efficient selective hydrogenation of acetylene to ethylene in excess ethylene. ACS Catal 2015; 5: 3717–25. 10.1021/acscatal.5b00700

[14] Wrasman CJ , Boubnov A, Riscoe AR et al. Synthesis of colloidal Pd/Au dilute alloy nanocrystals and their potential for selective catalytic oxidations. J Am Chem Soc 2018; 140: 12930–9. 10.1021/jacs.8b07515 30220200

[15] Marcinkowski MD , Darby MT, Liu J et al. Pt/Cu single-atom alloys as coke-resistant catalysts for efficient C-H activation. Nat Chem 2018; 10: 325–32. 10.1038/nchem.2915 29461520

[16] Giannakakis G , Flytzani-Stephanopoulos M, Sykes ECH. Single-atom alloys as a reductionist approach to the rational design of heterogeneous catalysts. Acc Chem Res 2019; 52: 237–47. 10.1021/acs.accounts.8b00490 30540456

[17] Sun G , Zhao ZJ, Mu R et al. Breaking the scaling relationship via thermally stable Pt/Cu single atom alloys for catalytic dehydrogenation. Nat Commun 2018; 9: 4454. 10.1038/s41467-018-06967-8 30367052 PMC6203812

[18] Wang A , Li J, Zhang T. Heterogeneous single-atom catalysis. Nat Rev Chem 2018; 2: 65–81. 10.1038/s41570-018-0010-1

[19] Jiao L , Jiang HL. Metal-organic-framework-based single-atom catalysts for energy applications. Chem 2019; 5: 786–804. 10.1016/j.chempr.2018.12.011

[20] Luo M , Guo S. Strain-controlled electrocatalysis on multimetallic nanomaterials. Nat Rev Mater 2017; 2: 17059. 10.1038/natrevmats.2017.59

[21] Wang X , Zhu Y, Vasileff A et al. Strain effect in bimetallic electrocatalysts in the hydrogen evolution reaction. ACS Energy Lett 2018; 3: 1198–204. 10.1021/acsenergylett.8b00454

[22] Furukawa H , Cordova KE, O’Keeffe M et al. The chemistry and applications of metal-organic frameworks. Science 2013; 341: 1230444. 10.1126/science.1230444 23990564

[23] Zhou HC , Kitagawa S. Meta-organic frameworks MOFs. Chem Soc Rev 2014; 43: 5415–8. 10.1039/C4CS90059F 25011480

[24] Li B , Wen HM, Cui Y et al. Emerging multifunctional metal-organic framework materials. Adv Mater 2016; 28: 8819–60. 10.1002/adma.201601133 27454668

[25] Jiao L , Wang Y, Jiang HL et al. Metal-organic frameworks as platforms for catalytic applications. Adv Mater 2018; 30: 1703663. 10.1002/adma.201703663 29178384

[26] Dhakshinamoorthy A , Li Z, Garcia H. Catalysis and photocatalysis by metal organic frameworks. Chem Soc Rev 2018; 47: 8134–72. 10.1039/C8CS00256H 30003212

[27] Islamoglu T , Goswami S, Li Z et al. Postsynthetic tuning of metal-organic frameworks for targeted applications. Acc Chem Res 2017; 50: 805–13. 10.1021/acs.accounts.6b00577 28177217

[28] Silva CG , Luz I, Xamena F et al. Water stable Zr-benzenedicarboxylate metal-organic frameworks as photocatalysts for hydrogen generation. Chem Eur J 2010; 16: 11133–8. 10.1002/chem.200903526 20687143

[29] Wu ZL , Wang CH, Zhao B et al. A semi-conductive copper-organic framework with two types of photocatalytic activity. Angew Chem Int Ed 2016; 55: 4938–42. 10.1002/anie.201508325 27079818

[30] Xu C , Liu H, Li D et al. Direct evidence of charge separation in a metal-organic framework: efficient and selective photocatalytic oxidative coupling of amines via charge and energy transfer. Chem Sci 2018; 9: 3152–8. 10.1039/C7SC05296K 29732097 PMC5916110

[31] Wu G , Huang J, Zang Y et al. Porous field-effect transistors based on a semiconductive metal-organic framework. J Am Chem Soc 2017; 139: 1360–3. 10.1021/jacs.6b08511 27794592

[32] Zhou T , Du Y, Borgna A et al. Post-synthesis modification of a metal-organic framework to construct a bifunctional photocatalyst for hydrogen production. Energy Environ Sci 2013; 6: 3229–34. 10.1039/c3ee41548a

[33] Kim D , Whang DR, Park SY. Self-healing of molecular catalyst and photosensitizer on metal-organic framework: robust molecular system for photocatalytic H_2_ evolution from water. J Am Chem Soc 2016; 138: 8698–701. 10.1021/jacs.6b04552 27356034

[34] Zhang T , Lin W. Metal-organic frameworks for artificial photosynthesis and photocatalysis. Chem Soc Rev 2014; 43: 5982–93. 10.1039/C4CS00103F 24769551

[35] An Y , Liu Y, An P et al. Ni^II^ coordination to an Al-based metal-organic framework made from 2-aminoterephthalate for photocatalytic overall water splitting. Angew Chem Int Ed 2017; 56: 3036–40. 10.1002/anie.201612423 28170148

[36] Yang SZ , Pattengale B, Kovrigin EL et al. Photoactive zeolitic imidazolate framework as intrinsic heterogeneous catalysts for light-driven hydrogen generation. ACS Energy Lett 2017; 2: 75–80. 10.1021/acsenergylett.6b00540

[37] Fu Y , Sun D, Chen Y et al. An amine-functionalized titanium metal-organic framework photocatalyst with visible-light-induced activity for CO_2_ reduction. Angew Chem Int Ed 2012; 51: 3364–7. 10.1002/anie.201108357 22359408

[38] Xu HQ , Hu J, Wang D et al. Visible-light photoreduction of CO_2_ in a metal-organic framework: boosting electron-hole separation via electron trap states. J Am Chem Soc 2015; 137: 13440–3. 10.1021/jacs.5b08773 26434687

[39] Zhang H , Wei J, Dong J et al. Efficient visible-light-driven carbon dioxide reduction by a single-atom implanted metal-organic framework. Angew Chem Int Ed 2016; 55: 14310–4. 10.1002/anie.201608597 27736031

[40] Wang Y , Huang NY, Shen JQ et al. Hydroxide ligands cooperate with catalytic centers in metal-organic frameworks for efficient photocatalytic CO_2_ reduction. J Am Chem Soc 2018; 140: 38–41. 10.1021/jacs.7b10107 29258308

[41] Wu LY , Mu YF, Guo XX et al. Encapsulating perovskite quantum dots in iron-based metal-organic frameworks (MOFs) for efficient photocatalytic CO_2_ reduction. Angew Chem Int Ed 2019; 58: 9491–5. 10.1002/anie.201904537 31066965

[42] Li N , Liu J, Liu JJ et al. Adenine components in biomimetic metal-organic frameworks for efficient CO_2_ photoconversion. Angew Chem Int Ed 2019; 58: 5226–30. 10.1002/anie.201814729 30656814

[43] Xia Z , He C, Wang X et al. Modifying electron transfer between photoredox and organocatalytic units via framework interpenetration for β-carbonyl functionalization. Nat Commun 2017; 8: 361. 10.1038/s41467-017-00416-8 28842552 PMC5572462

[44] Zhang Y , Zhang Y, Guo J et al. Tunable chiral metal organic frameworks toward visible light-driven asymmetric catalysis. Sci Adv 2017; 3: e1701162. 10.1126/sciadv.1701162 28835929 PMC5562422

[45] Xiao JD , Jiang HL. Metal-organic frameworks for photocatalysis and photothermal catalysis. Acc Chem Res 2019; 52: 356–66. 10.1021/acs.accounts.8b00521 30571078

[46] Wang C , deKrafft KE, Lin W. Pt nanoparticles@photoactive metal-organic frameworks: efficient hydrogen evolution via synergistic photoexcitation and electron injection. J Am Chem Soc 2012; 134: 7211–4. 10.1021/ja300539p 22486151

[47] Shen L , Luo M, Huang L et al. A clean and general strategy to decorate a titanium metal-organic framework with noble-metal nanoparticles for versatile photocatalytic applications. Inorg Chem 2015; 54: 1191–3. 10.1021/ic502609a 25594784

[48] Fang X , Shang Q, Wang Y et al. Single Pt atoms confined into a metal-organic framework for efficient photocatalysis. Adv Mater 2018; 30: 1705112. 10.1002/adma.201705112 29315871

[49] Zuo Q , Liu T, Chen C et al. Ultrathin metal-organic framework nanosheets with ultrahigh loading of single Pt atoms for efficient visible-light-driven photocatalytic H_2_ evolution. Angew Chem Int Ed 2019; 58: 10198–203. 10.1002/anie.201904058 31107580

[50] Hu Z , Peng Y, Kang Z et al. A modulated hydrothermal (MHT) approach for the facile synthesis of UiO-66-type MOFs. Inorg Chem 2015; 54: 4862–8. 10.1021/acs.inorgchem.5b00435 25932655

[51] Hu P , Morabito JV, Tsung CK. Core-shell catalysts of metal nanoparticle core and metal-organic framework shell. ACS Catal 2014; 4: 4409–19. 10.1021/cs5012662

[52] Yang Q , Xu Q, Jiang HL. Metal-organic frameworks meet metal nanoparticles: synergistic effect for enhanced catalysis. Chem Soc Rev 2017; 46: 4774–808. 10.1039/C6CS00724D 28621344

[53] Chen L , Huang B, Qiu X et al. Seed-mediated growth of MOF-encapsulated Pd@Ag core-shell nanoparticles: toward advanced room temperature nanocatalysts. Chem Sci 2016; 7: 228–33. 10.1039/C5SC02925B 28758001 PMC5515064

[54] Li X , Zhang B, Tang L et al. Cooperative multifunctional catalysts for nitrone synthesis: platinum nanoclusters in amine-functionalized metal-organic frameworks. Angew Chem Int Ed 2017; 56: 16371–4. 10.1002/anie.201710164 29065244

[55] Zhao M , Yuan K, Wang Y et al. Metal-organic frameworks as selectivity regulators for hydrogenation reactions. Nature 2016; 539: 76–80. 10.1038/nature19763 27706142

[56] Ge J , He DS, Chen W et al. Atomically dispersed Ru on ultrathin Pd nanoribbons. J Am Chem Soc 2016; 138: 13850–3. 10.1021/jacs.6b09246 27740759

[57] Tao F , Grass ME, Zhang Y et al. Reaction-driven restructuring of Rh-Pd and Pt-Pd core-shell nanoparticles. Science 2008; 322: 932–4. 10.1126/science.1164170 18845713

[58] Duchesne PN , Li ZY, Deming CP et al. Golden single-atomic-site platinum electrocatalysts. Nat Mater 2018; 17: 1033–9. 10.1038/s41563-018-0167-5 30250176

[59] Chao T , Luo X, Chen W et al. Atomically dispersed copper-platinum dual sites alloyed with palladium nanorings catalyze the hydrogen evolution reaction. Angew Chem Int Ed 2017; 56: 16047–51. 10.1002/anie.201709803 29063649

[60] Tedsree K , Li T, Jones S et al. Hydrogen production from formic acid decomposition at room temperature using a Ag-Pd core-shell nanocatalyst. Nat Nanotechnol 2011; 6: 302–7. 10.1038/nnano.2011.42 21478867

