# Supplementary material for: Precise fabrication of single-atom alloy co-catalyst with optimal charge state for enhanced photocatalysis
Source: Natl Sci Rev. 2020 Sep 3;8(1):nwaa224. doi: 10.1093/nsr/nwaa224 (PMC8288370; doi:10.1093/nsr/nwaa224)
Supplement: nwaa224_Supplemental_Files [file nwaa224_supplemental_files.doc]

**Supporting Information**

**Precise fabrication of single-atom alloy co-catalyst with optimal charge state for enhanced photocatalysis**

Yating Pan^1^, Yunyang Qian^1^, Xusheng Zheng^2^, Sheng-Qi Chu^3^, Yijun Yang^4^, Chunmei Ding^5^, Xi Wang^4^, Shu-Hong Yu^1^ and Hai-Long Jiang^1,*^

^1^Hefei National Laboratory for Physical Sciences at the Microscale, CAS Key Laboratory of Soft Matter Chemistry, Department of Chemistry, University of Science and Technology of China, Hefei, Anhui 230026, P.R. China

^2^National Synchrotron Radiation Laboratory (NSRL), University of Science and Technology of China, Hefei, Anhui 230029, P.R. China

^3^Beijing Synchrotron Radiation Facility, Institute of High Energy Physics, Chinese Academy of Sciences, Beijing 100049, P.R. China

^4^Key Laboratory of Luminescence and Optical Information, Ministry of Education, Department of Physics, School of Science, Beijing Jiaotong University, Beijing 100044, P.R. China

^5^Dalian National Laboratory for Clean Energy, State Key Laboratory of Catalysis, Dalian Institute of Chemical Physics, Chinese Academy of Sciences, Dalian 116023, P.R. China

*E-mail: [jianglab@ustc.edu.cn](mailto:jianglab@ustc.edu.cn)

**Section 1. Materials and Equipments**

All chemicals were obtained from commercial sources and used without further purification. Powder X-ray diffraction patterns (XRD) were obtained on a Japan Rigaku MiniFlex 600 equipped with graphite-monochromated Cu K*α* radiation (*λ* = 1.54178 Å). The Pd and Pt contents were quantified by an Optima 7300 DV inductively coupled plasma atomic emission spectrometer (ICP-AES). The transmission electron microscopy (TEM) images were acquired on a JEOL JEM-2100F field-emission transmission electron microscope. The high-resolution TEM (HRTEM) images, elemental line-scanning spectra and high-angle annular dark-field scanning transmission electron microscopy (HAADF-STEM) observation were conducted on a JEOL JEM-ARM200F TEM/STEM with a spherical aberration corrector (the HRTEM image and elemental line-scanning spectra were obtained at Beijing Jiaotong University, P. R. China). Field-emission scanning electron microscopy (FE-SEM) was carried out with a field S3 emission scanning electron microanalyzer (Zeiss Supra 40 scanning electron microscope at an acceleration voltage of 5 kV). X-ray photoelectron spectroscopy (XPS) measurements were conducted at the BL10B beamline in the National Synchrotron Radiation Laboratory (NSRL, Hefei, P. R. China) using monochromated Mg K*α* radiation (*hν* = 1253.6 eV) as the excitation source. UV-Vis absorption spectrum was recorded on a Shimadzu UV-2700 spectrophotometer with BaSO_4_ as a reference. Photoluminescence (PL) spectra (excited at 360 nm) were measured with a PerkinElmer LS 55 Fluorescence Spectrometer. The catalytic reaction products were analyzed and identified by gas chromatography (Shimadzu GC-2014).

**Section 2. Preparation of catalysts**

**Preparation of UiO-66-NH_2_.** The UiO-66-NH_2_ was synthesized with some modifications based on the previous report [1]. Generally, 186 mg ZrCl_4_ and 145 mg 2-aminoterephthalic acid (ATA) were ultrasonically dissolved in 50 mL DMF containing 6 mL HOAc and 100 μL H_2_O in a 100 mL round-bottom flask. Then, the mixture was reacted in oil bath at 120 °C for 24 h. The product was harvested by centrifugation and washed with DMF and methanol for several times, followed by dried at 60 °C under vacuum overnight.

**Preparation of Pd_10_/UiO-66-NH_2_.** Generally, 15 mg Pd^2+^/UiO-66-NH_2_ (obtained as described in the preparation of Pd@Pt/UiO-66-NH_2_) was dispersed in 5 mL MeOH by ultrasonication. Then, 5 mg NH_3_BH_3_ was added and stirred for 1h. After that, the product was isolated by centrifugation and washed by MeOH for several times, and dried at 60 °C under vacuum overnight.

**Preparation of Pt_1_/UiO-66-NH_2_.** A certain amount (Pt/UiO-66-NH_2_ = 0.1 wt%) of H_2_PtCl_6_ aqueous solution was added into 100 mg UiO-66-NH_2_ in a porcelain crucible. Then, the mixture was dispersed in 200 μL MeOH, stirred with a glass rod rapidly and heated at 80 °C to evaporate the solution. This procedure was repeated twice and the obtained solid was dried in 85 °C oven for 15 min to get Pt^2+^/UiO-66-NH_2_. The rest of the preparation procedure for Pt_1_/UiO-66-NH_2_ was similar to that for Pd_10_/UiO-66-NH_2_ except for the replacement of Pd^2+^/UiO-66-NH_2_ with Pt^2+^/UiO-66-NH_2_.

**Preparation of Pd_10_+Pt_1_/UiO-66-NH_2_.** Typically, 100 mg Pd_10_/UiO-66-NH_2_ was dispersed in 200 μL MeOH and certain amount of H_2_PtCl_6_ aqueous solution was added (the mass ratio of Pd/Pt = 10/1). Then, the mixture was rapidly stirred with a glass rod and heated at 80 °C to evaporate the solution. After repeating this procedure twice, the solid was dried in 85 °C oven for 15 min. The remaining process for Pd_10_+Pt_1_/UiO-66-NH_2_ preparation was similar to that for Pd_10_/UiO-66-NH_2_ except for the replacement of Pd^2+^/UiO-66-NH_2_ with the obtained solid.

**Preparation of Pd_10_Pt_1_/UiO-66-NH_2_.** Generally, 100 mg UiO-66-NH_2_ was put in a porcelain crucible and a certain amount of 50 mg/mL Pd(NO_3_)_2_ and H_2_PtCl_6_ mixed aqueous solution (Pd/Pt = 10/1 wt%) was added. Then, 200 μL MeOH was added into the mixture and rapidly stirred with a glass rod and heated at 80 °C to evaporate the solution. This procedure was repeated twice and the obtained solid was dried in 85 °C oven for 15 min. The obtained powder (15 mg) was dispersed in 5 mL MeOH by ultrasonication. Then, 5 mg NH_3_BH_3_ was added and stirred for 1 h. After that, the product was isolated by centrifugation and washed by MeOH for several times, finally dried at 60 °C under vacuum overnight.

**Section 3. Electrochemical measurements**

**Photoelectrochemical measurements.** Under the conditions of a 300 W Xenon lamp (LX-300F, Japan) with UV cut-off filter (> 380 nm) as light source and a 0.1 M Na_2_SO_4_ solution as electrolyte, the photoelectrochemical measurements were performed on a CHI 760E electrochemical work station (Chenhua Instrument, Shanghai, China) with a standard three-electrode system with the photocatalyst-coated ITO as the working electrode, Pt plate as the counter electrode, and Ag/AgCl as a reference electrode. The working electrodes were prepared by dropping a 200 µL suspension (prepared by dispersing 2 mg catalyst into 10 µL Nafion and 3 mL ethanol mixed solution) onto the surface of an ITO plate with an exposed area of 1.0 × 1.0 cm^2^. A bias potential of + 0.5 V was applied in this measurement process.

**Electrochemical impedance spectroscopy.** The electrochemical impedance spectroscopy was performed on the Zahner Zennium electrochemical work station in a standard three-electrode system: the working electrode (the photocatalyst-coated glassy carbon electrode), counter electrode (Pt plate), and a reference electrode (Ag/AgCl), with a 0.1 M Na_2_SO_4_ aqueous solution as electrolyte. The samples (2 mg) was dispersed into 10 µL 5 wt% Nafion and 3 mL ethanol mixed solution, and the working electrode was prepared by dropping the suspension (30 µL) onto the surface of the glassy carbon electrode. Then, the EIS measurement was performed with a bias potential of -1.3 V in the dark.

**Cathodic polarization curves.** The cathodic polarization curves were collected on a CHI 760E electrochemical work station (Chenhua Instrument, Shanghai, China) with a standard three-electrode system (the photocatalyst-coated ITO as the working electrode, Pt plate as the counter electrode, and Ag/AgCl as a reference electrode) and 0.5 M Na_2_SO_4_ aqueous solution as electrolyte. The working electrodes were prepared by dropping a 150 µL suspension (prepared by dispersing 2 mg catalyst into 10 µL Nafion and 1 mL ethanol mixed solution) onto the surface of an ITO plate with an exposed area of 2.0 × 2.0 cm^2^. A bias potential of -0.6 V to -0.1 eV was applied in this measurement process and the system was exposed to dark and light irradiation every 0.05 eV alternately (a 300 W Xenon lamp (LX-300F, Japan) with UV cut-off filter (> 380 nm) as light source).

**Section 4. Computational methods**

DFT calculations were performed to simulate the charge state of Pd@Pt NPs by using Vienna ab initio package software [2]. The projector augmented wave (PAW) model with the generalized gradient approximation (GGA) and Perdew-Burke-Ernzerhof (PBE) functions were applied [3, 4]. The plane-wave basis with energy cut-off point of 450 eV was adopted to extend the electron wave function. The force and energy convergence criterion was set to 10^-5^ eV and 0.02 eV/Å, respectively. Pd (111) surface was modeled by 3×3 slab system with four layers and 15 Å vacuum region to the surface to ensure decoupling between next slabs. The Pd@Pt NPs was obtained by Pd (111) with surface Pd atoms being occupied/covered by Pt atoms/layers according to the Pd/Pt molar ratio (Supplementary Tables 1 and 2). The 3×3×1 Gamma centred k-point were adopted for the cell in the Gibbs free energy calculation. The free energy of adsorbed H (ΔG_H*_) were taken as: ΔG_H*_ = ΔE_H*_ + ΔE_ZPE_ – TΔS, where ΔE_H*_ is the adsorption energy of H species, ΔE_ZPE_ and ΔS are the difference of zero point energy and entropy between H* and the gas phase, respectively and T is the system temperature (298.15 K). Dipole correction was used in standard free energy of HER on the SAA.

**Supplementary Figure 1.** Powder XRD patterns of catalysts before reaction. Powder XRD patterns of simulated and experimental UiO-66-NH_2_, Pt_1_/UiO-66-NH_2_, Pd_10_/UiO-66-NH_2_, Pd_10_+Pt_1_/UiO-66-NH_2_, Pd_10_Pt_1_/UiO-66-NH_2_, and Pd_10_@Pt_x_/UiO-66-NH_2_ (x = 0.3, 1, 5, 10).

**Supplementary Figure 2.** TEM observation of Pd_10_/UiO-66-NH_2_. (a) TEM image of Pd_10_/UiO-66-NH_2_ and (b) the corresponding size distribution for Pd NPs in Pd_10_/UiO-66-NH_2_. The TEM results indicate that most Pd NPs are well dispersed and might be stabilized onto the MOF surface.

**Supplementary Figure 3.** TEM observation of Pd_10_@Pt_0.3_/UiO-66-NH_2_. (a) TEM image of Pd_10_@Pt_0.3_/UiO-66-NH_2_ and (b) the corresponding size distribution for Pd_10_@Pt_0.3_ NPs in Pd_10_@Pt_0.3_/UiO-66-NH_2_.

**Supplementary Figure 4.** TEM observation of Pd_10_@Pt_1_/UiO-66-NH_2_. (a) TEM image of Pd_10_@Pt_1_/UiO-66-NH_2_ and (b) the corresponding size distribution for Pd_10_@Pt_1_ NPs in Pd_10_@Pt_1_/UiO-66-NH_2_.

**Supplementary Figure 5.** TEM observation of Pd_10_@Pt_5_/UiO-66-NH_2_. (a) TEM image of Pd_10_@Pt_5_/UiO-66-NH_2_ and (b) the corresponding size distribution for Pd_10_@Pt_5_ NPs in Pd_10_@Pt_5_/UiO-66-NH_2_.


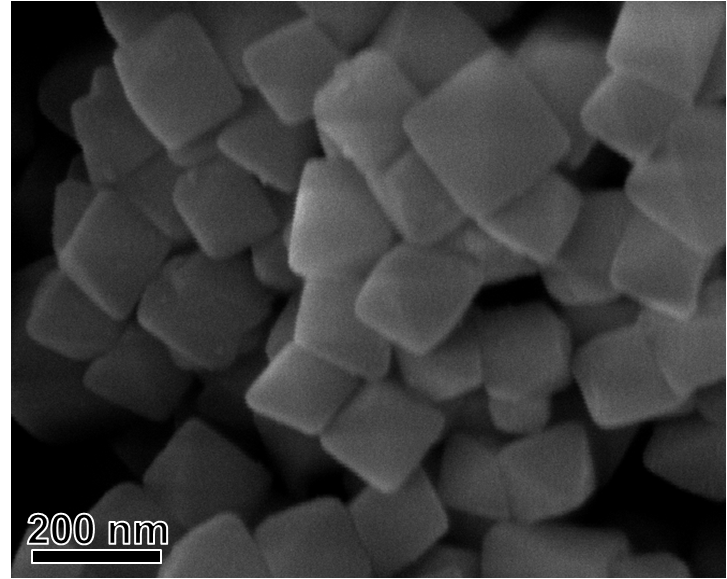


**Supplementary Figure 6.** SEM observation of Pd_10_/UiO-66-NH_2_.


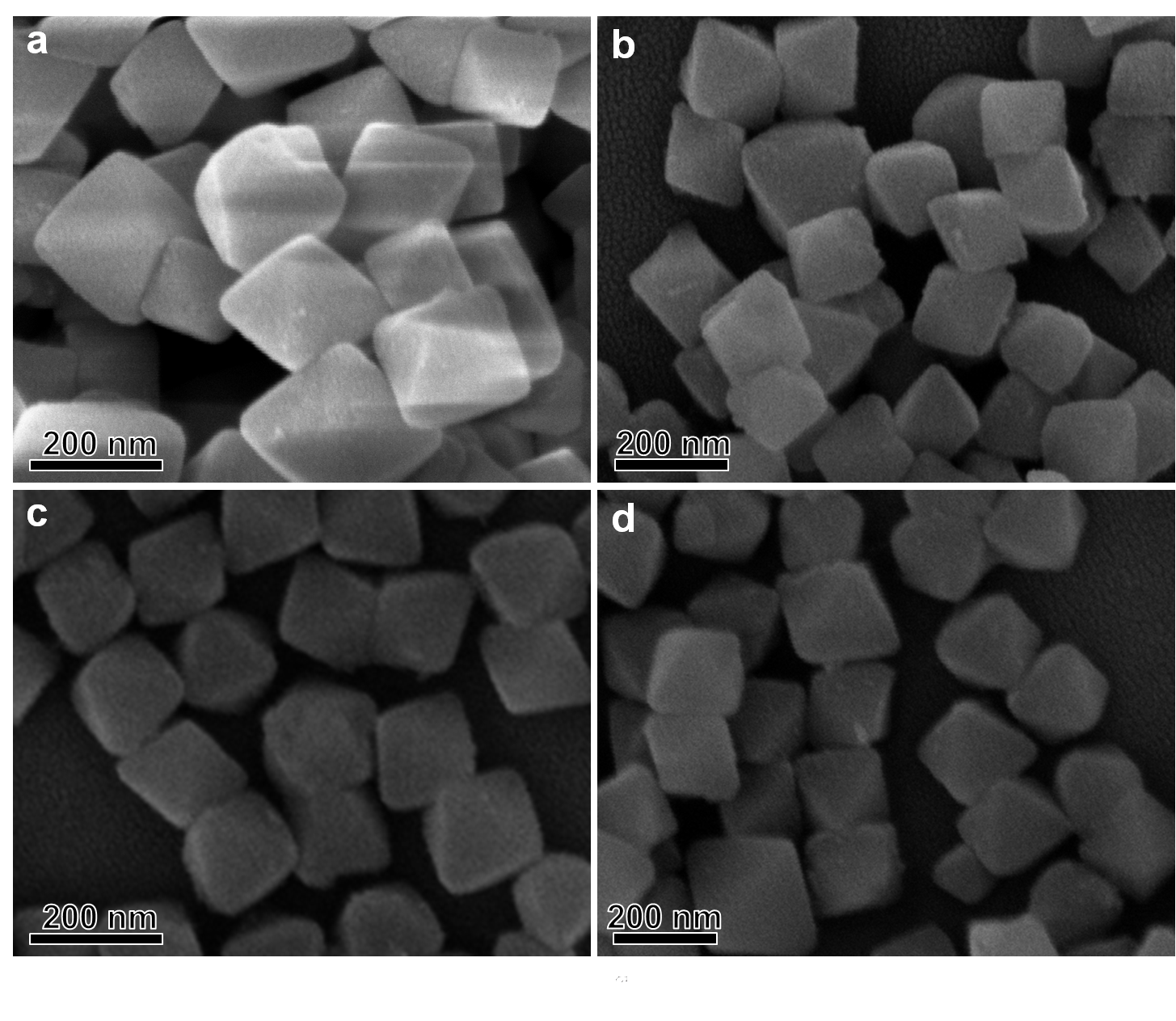


**Supplementary Figure 7.** SEM observation of Pd_10_@Pt*_x_*/UiO-66-NH_2_. SEM images of (a) Pd_10_@Pt_0.3_/UiO-66-NH_2_, (b) Pd_10_@Pt_1_/UiO-66-NH_2_, (c) Pd_10_@Pt_5_/UiO-66-NH_2_ and (d) Pd_10_@Pt_10_/UiO-66-NH_2_.


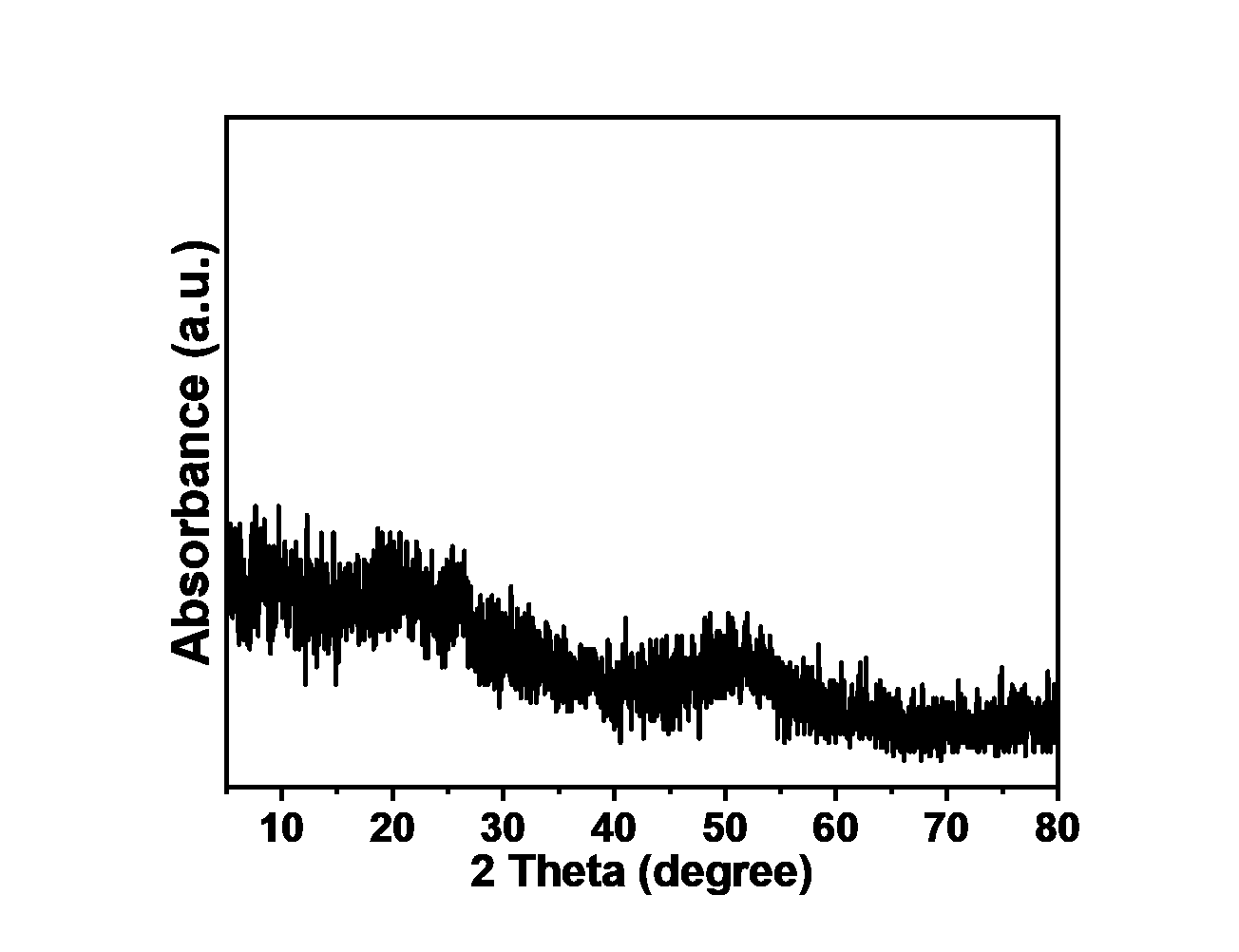


**Supplementary Figure 8.** Powder XRD pattern. Powder XRD pattern of Pd_10_@Pt_10_/UiO-66-NH_2_ after being treated with H_3_PO_4_ to remove the MOF.

**Supplementary Figure 9.** TEM observation of Pd_10_@Pt_10_/UiO-66-NH_2_ (treated with H_3_PO_4_). (a) TEM image of Pd_10_@Pt_10_/UiO-66-NH_2_, after being treated with H_3_PO_4_ to remove the MOF and (b) the corresponding size distribution for Pd_10_@Pt_10_ NPs.

TEM observation shows that the residual Pd_10_@Pt_10_ NPs are able to remain similar sizes and morphology to them in Pd_10_@Pt_10_/UiO-66-NH_2_.


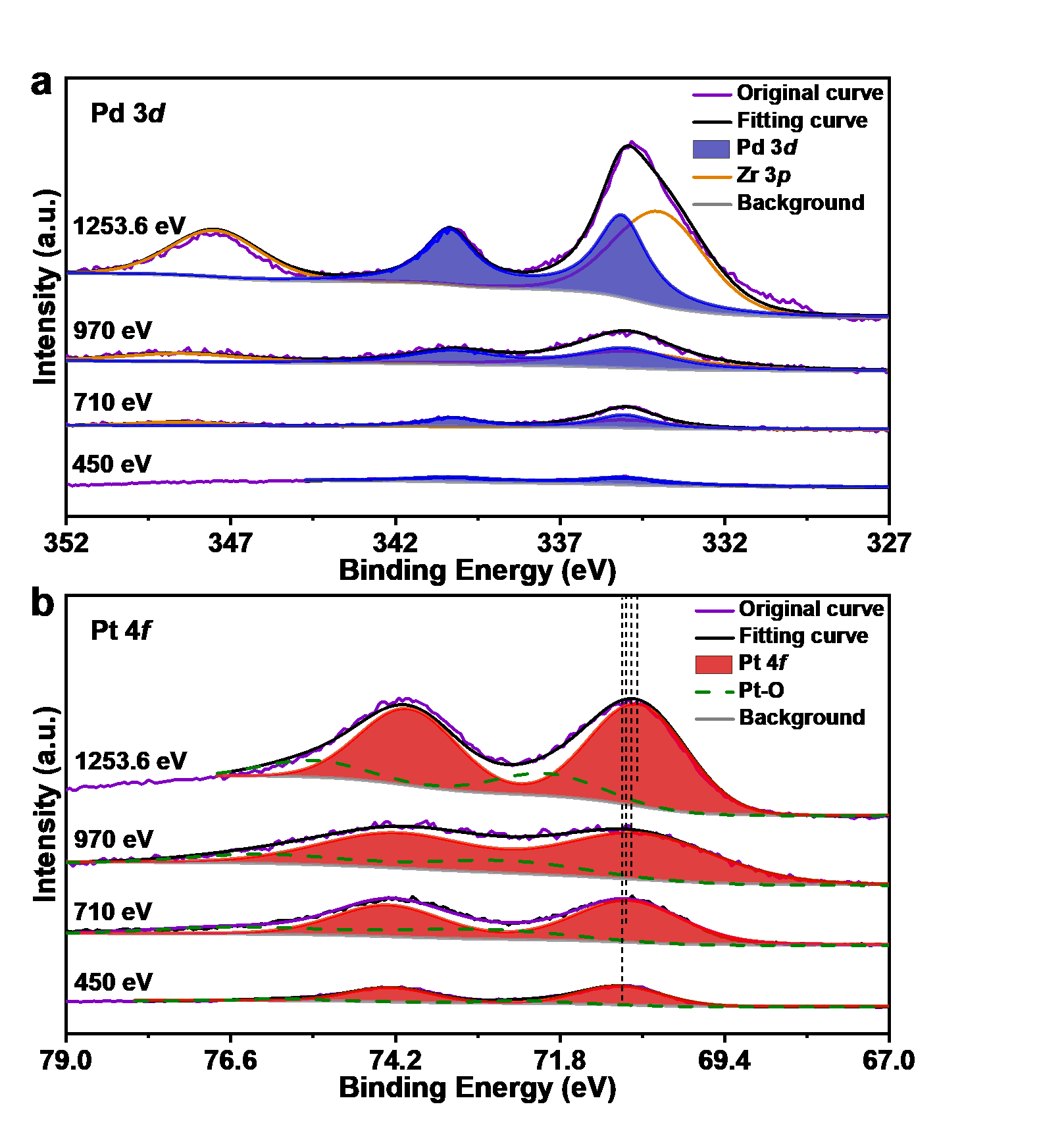


**Supplementary Figure 10.** SRPES spectra. (a) Pd 3*d* and (b) Pt 4*f* SRPES spectra for Pd_10_@Pt_10_/UiO-66-NH_2_ (after being treated with H_3_PO_4_) with different photon energies.

Peaks around 334.1 and 347.6 eV are the 3*p*_3/2_ and 3*p*_1/2_ peaks of residual Zr after acid treatment. Furthermore, weak signals at *ca.* 72.7 and 76.0 eV suggest that the formation Pt-O bond might occur to some Pt surface due to the oxidation [5, 6].When the probe depth gets close to the Pd core as photon energy increases, the Pt binding energy appears a negative shift, which suggests the enhanced charge redistribution between Pd and Pt.

**Supplementary Figure 11.** Elemental line-scanning spectra. Elemental line-scanning spectra of Pd_10_@Pt_10_ particle (stabilized by UiO-66-NH_2_) related to Figure 2d.


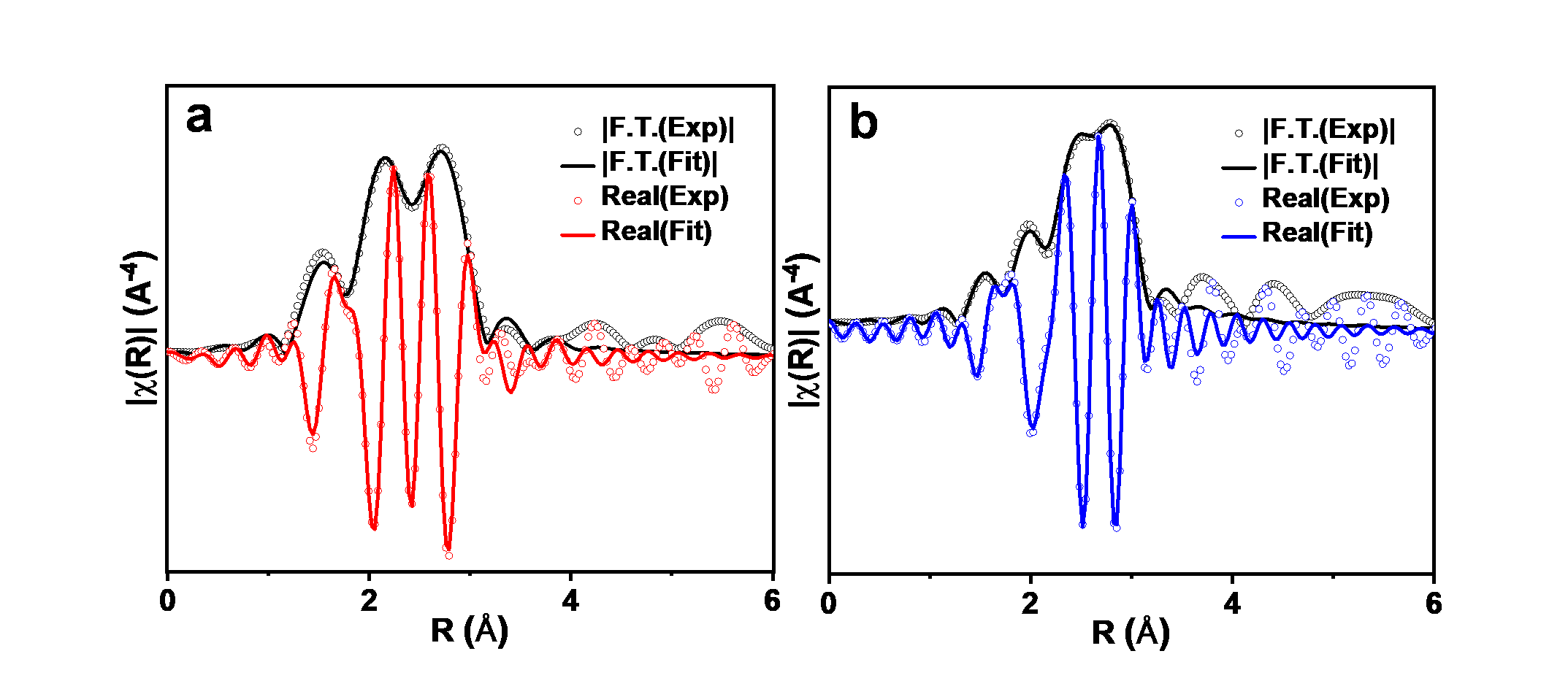


**Supplementary Figure 12.** EXAFS spectra fitting. (a) Fitting results of the EXAFS spectra of Pd_10_@Pt_1_/UiO-66-NH_2_ and (b) Pd_10_@Pt_10_/UiO-66-NH_2_.


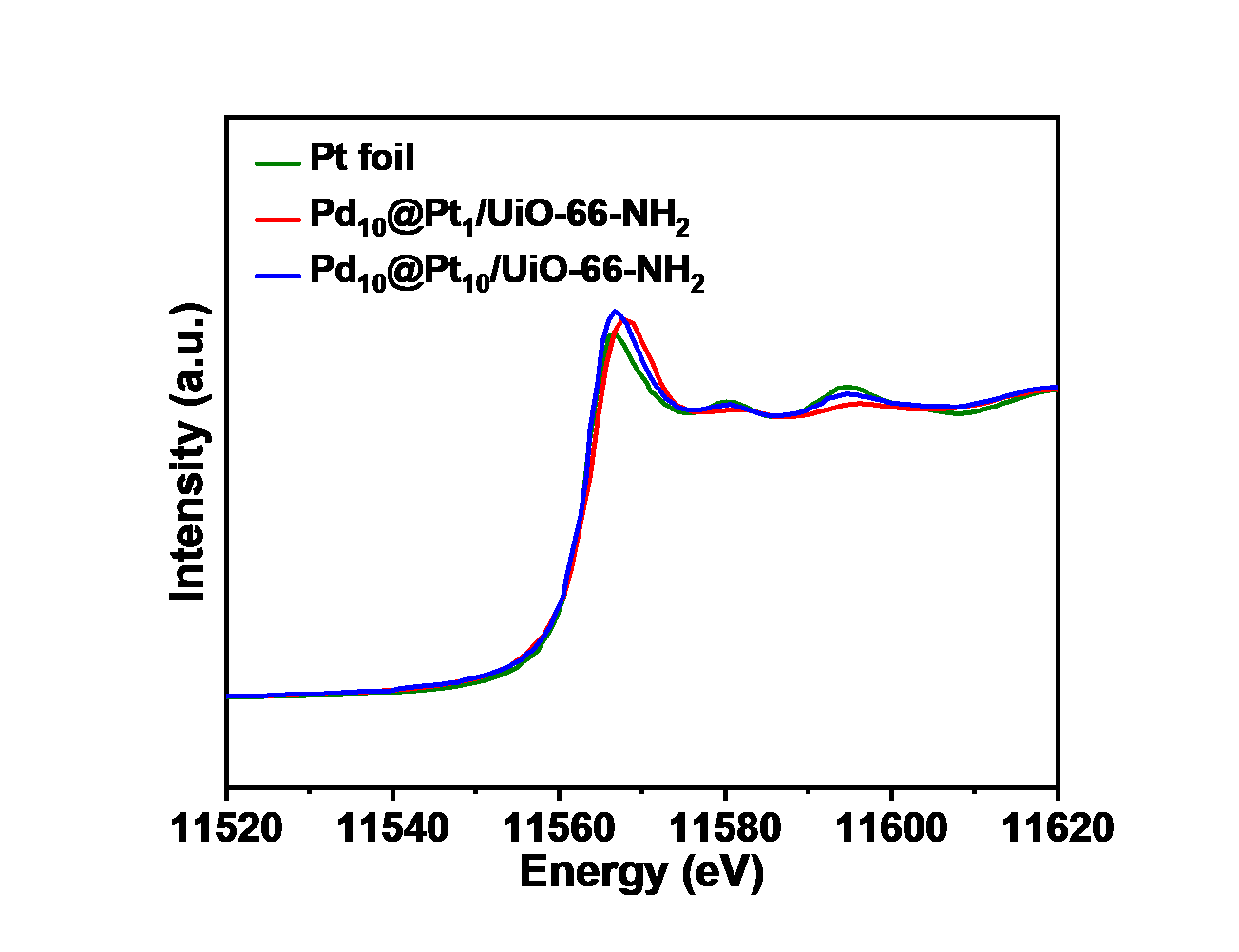


**Supplementary Figure 13.** XANES spectra. XANES spectra of Pd_10_@Pt_1_/UiO-66-NH_2_ and Pd_10_@Pt_10_/UiO-66-NH_2_ for Pt L_3_-edge.

**Supplementary Figure 14.** Work function. The work function of Pd, Pd@Pt_SAA_, core-shell Pd@Pt_3 layers_, and Pt simulated by density-functional theory (DFT) calculations.

The Pd shows lower work function than Pt according to the DFT calculations. To balance the Fermi distribution of electrons in Pd@Pt NPs, electrons tend to leave Pd and travel to Pt at their interface, resulting in charge redistribution, which affects the Pt surface charge state. The more enhanced charge redistribution effect and the most electron-rich Pt in SAA structure may result in the lower work function of Pd@Pt_SAA_ than core-shell Pd@Pt_3 layers_.

**Supplementary Figure 15.** Bader charge and binding energy shift. The Bader charge of each Pt atom (black line) and the binding energy shift of Pt 4*f*_7/2_ (red line) *vs.* the number of Pt layer.

Both the binding energy shift of Pt 4*f*_7/2_ and the Bader charge of Pt atoms from differential charge density of Pd@Pt NPs are able to reflect charge transfer between Pd and Pt. Herein, the Bader charge of Pt atoms is employed to simulate the XPS shift. The Bader charge of each Pt atom and the binding energy shift of Pt 4*f*_7/2_ along with the Pt layer thickness show similar slopes, indicating the consistent charge redistribution trend in simulation and experiment (for specific values, see Supplementary Tables 5 and 6).


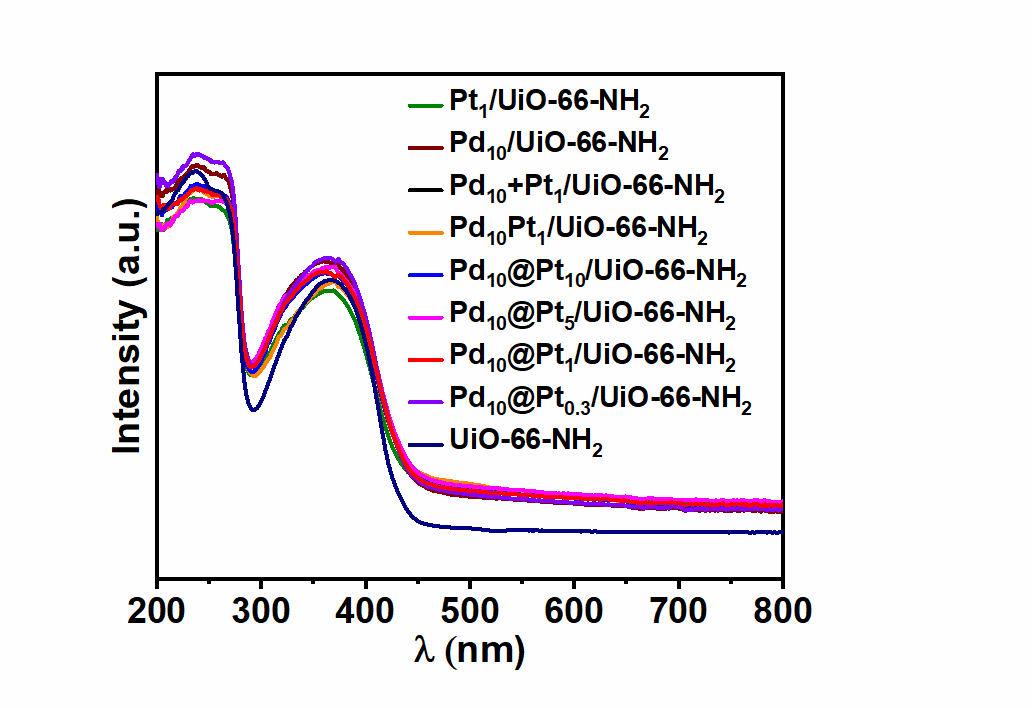


**Supplementary Figure 16.** UV-Vis absorption spectra. UV-Vis absorption spectra of Pt_1_/UiO-66-NH_2_, Pd_10_/UiO-66-NH_2_, Pd_10_+Pt_1_/UiO-66-NH_2_, Pd_10_Pt_1_/UiO-66-NH_2_ Pd_10_@Pt_x_/UiO-66-NH_2_ and UiO-66-NH_2_.

All Pd_10_@Pt_x_/UiO-66-NH_2_ present similar UV-Vis absorption peaks indicating their comparable light absorption behavior. The close contact between UiO-66-NH_2_ and metal nanoparticles leads to significant high scattering and interband electronic transition at long wavelengths (450-800 nm) [7], in reference to UiO-66-NH_2_.

**Supplementary Figure 17.** TEM observation and related Pt sizes for Pt_1_/UiO-66-NH_2_. (a) TEM image of Pt_1_/UiO-66-NH_2_ and (b) the corresponding size distribution for Pt_1_ NPs in Pt_1_/UiO-66-NH_2_.

**Supplementary Figure 18.** TEM observation of Pd_10_+Pt_1_/UiO-66-NH_2_. (a) TEM image (inset: the corresponding size distribution for metal NPs) and (b) enlarged TEM image of Pd_10_+Pt_1_/UiO-66-NH_2_.

**Supplementary Figure 19.** TEM observation of Pd_10_Pt_1_/UiO-66-NH_2_. (a) TEM image (inset: the corresponding size distribution for PdPt NPs) and (b) enlarged TEM image of Pd_10_Pt_1_/UiO-66-NH_2_.


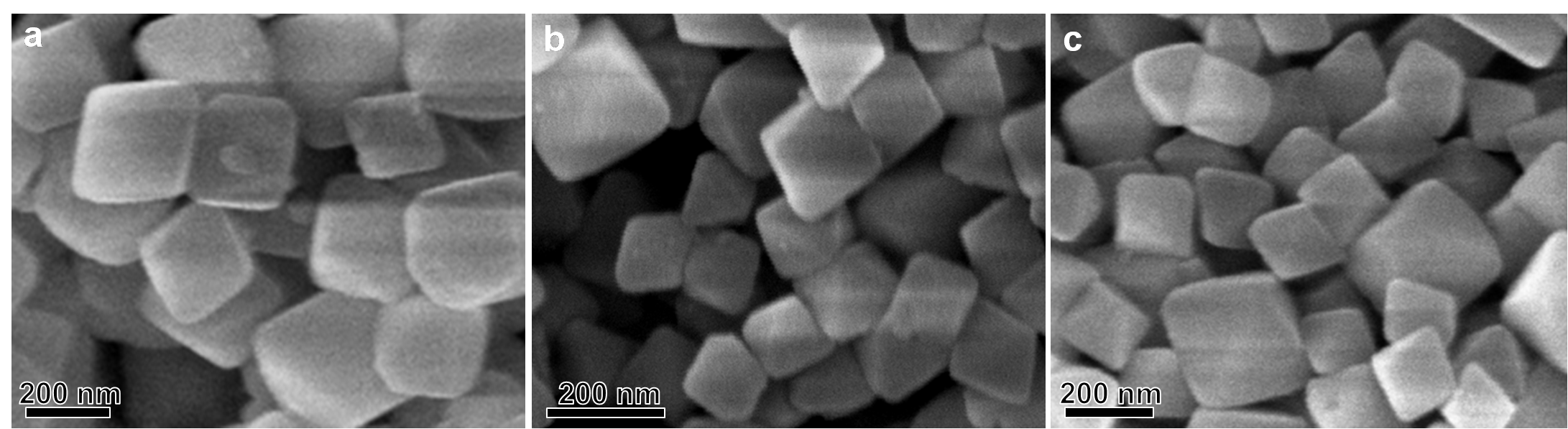


**Supplementary Figure 20.** SEM observation. SEM images of (a) Pt_1_/UiO-66-NH_2_, (b) Pd_10_+Pt_1_/UiO-66-NH_2_ and (c) Pd_10_Pt_1_/UiO-66-NH_2_.

**Supplementary Figure 21.** Calculated free energy diagram for photocatalytic H_2_ production. ΔG_H*_ on Pt and Pd in the Pd_10_@Pt_1_ SAA structure.

Based on the calculated Gibbs free energy (ΔG_H*_) values of Pt and Pd atoms in the Pd_10_@Pt_1_ SAA structure, the lower ΔG_H*_ on Pt than Pd suggests that, in the SAA structure, Pt is more favorable active site to Pd for hydrogen production.

**Supplementary Figure 22.** Powder XRD patterns of catalysts after reaction. Powder XRD patterns for different catalysts after photocatalytic reaction.


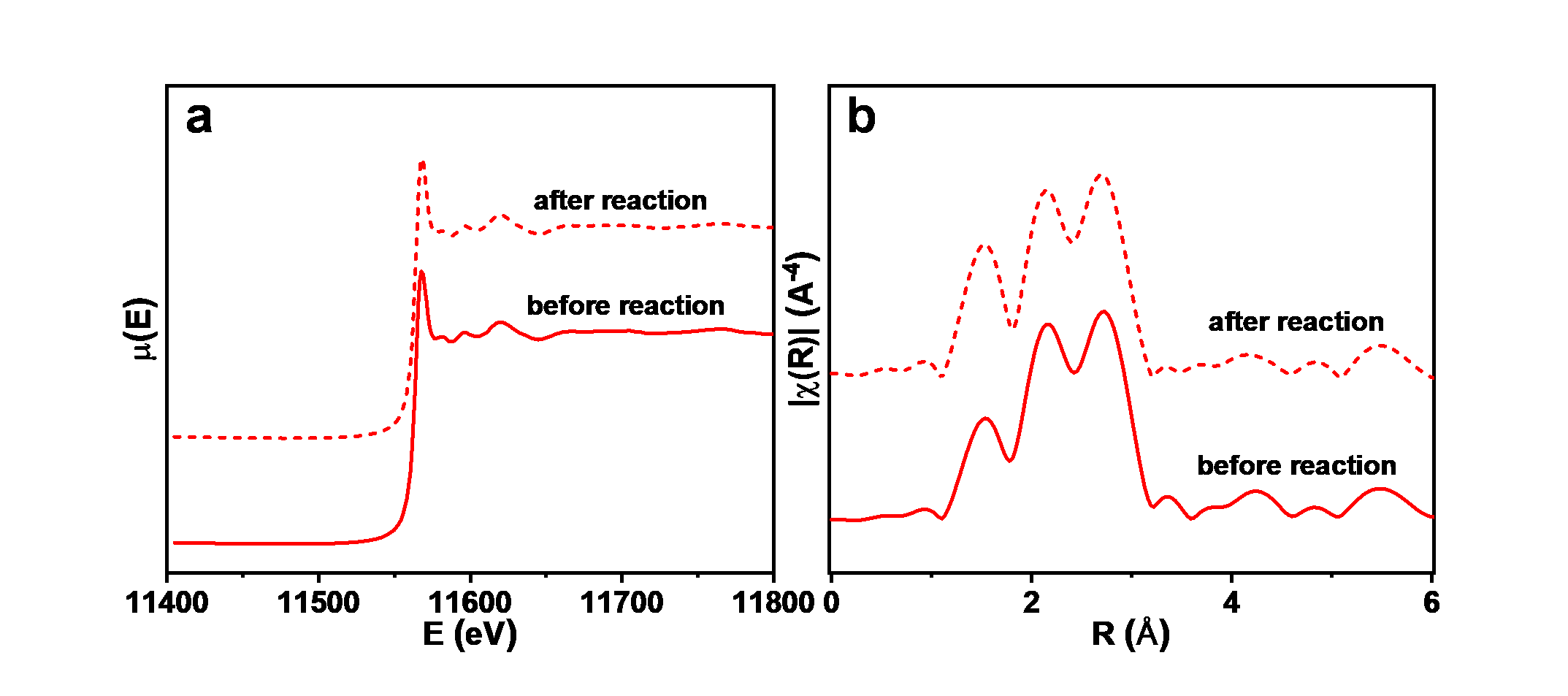


**Supplementary Figure 23.** XANES sand FT-EXAFS spectra for Pd_10_@Pt_1_/UiO-66-NH_2_ before and after reaction. (a) Pt L_3_-edge XANES spectra and (b) Fourier transformed (FT) *k*3-weighted χ(*k*)-function of the Pt L_3_-edge EXAFS spectra for Pd_10_@Pt_1_/UiO-66-NH_2_ before and after reaction.

**Supplementary Figure 24.** TEM observation of Pd_10_@Pt_1_/UiO-66-NH_2_ after reaction. (a) TEM image (inset: the corresponding size distribution for metal NPs) and (b) enlarged TEM image of Pd_10_@Pt_1_/UiO-66-NH_2_ after reaction.

**Supplementary Figure 25.** Long-term test. Time-course of the photocatalytic H_2_ production of Pd_10_@Pt_1_/UiO-66-NH_2_.

**Supplementary Figure 26.** EIS Nyquist plots. EIS Nyquist plots for different samples.

**Supplementary Figure 27.** Cathodic polarization curves. The cathodic polarization curves of Pd_10_/UiO-66-NH_2_, Pt_1_/UiO-66-NH_2_, Pd_10_@Pt_1_/UiO-66-NH_2_ and Pd_10_@Pt_10_/UiO-66-NH_2_ under dark and light irradiation (inset: the enlarged image around -0.45 to -0.4 V potential).

The cathodic polarization curves indicate Pd_10_@Pt_1_/UiO-66-NH_2_ has lower over potential than other catalysts. The slight increase in photocurrent response under illumination for Pd_10_@Pt_1_/UiO-66-NH_2_ (see inset) suggests that its charge separation and transfer are pronounced, in reference to other catalysts.

**Supplementary Table 1.** ICP-AES results.

|  | Pd  (wt%) | | Pt  (wt%) | | Pd/Pt  mass ratio | Pd/Pt  molar ratio |
| --- | --- | --- | --- | --- | --- | --- |
| Pd_10_/UiO-66-NH_2_ | | 1.70 | 0.00 | | / | / |
| Pt_1_/UiO-66-NH_2_ | | 0.00 | | 0.07 | / | / |
| Pd_10_+Pt_1_/UiO-66-NH_2_ | | 1.75 | | 0.09 | 10 : 0.7 | 97.2 : 2.8 |
| Pd_10_Pt_1_/UiO-66-NH_2_ | | 1.72 | | 0.09 | 10 : 0.5 | 97.3 : 2.7 |
| Pd_10_@Pt_0.3_/UiO-66-NH_2_ | | 1.61 | | 0.02 | 10 : 0.1 | 99.5 : 0.5 |
| Pd_10_@Pt_1_/UiO-66-NH_2_ | | 1.69 | | 0.12 | 10 : 0.7 | 96.2 : 3.8 |
| Pd_10_@Pt_5_/UiO-66-NH_2_ | | 1.64 | | 0.34 | 10 : 2.1 | 88.5 : 11.5 |
| Pd_10_@Pt_10_/UiO-66-NH_2_ | | 1.70 | | 1.09 | 10 : 5.5 | 70.0 : 30.0 |

**Supplementary Table 2.** The average diameter and Pt layers of metal NPs in Pd_10_/UiO-66-NH_2_ and Pd_10_@Pt_x_/UiO-66-NH_2_.

| Cat | Diameter (nm) | Pt layers^a^ |
| --- | --- | --- |
| Pd_10_/UiO-66-NH_2_ | 4.01 | / |
| Pd_10_@Pt_0.3_/UiO-66-NH_2_ | 4.07 | / |
| Pd_10_@Pt_1_/UiO-66-NH_2_ | 4.31 | / |
| Pd_10_@Pt_5_/UiO-66-NH_2_ | 4.68 | 1.5 |
| Pd_10_@Pt_10_/UiO-66-NH_2_ | 5.10 | 2.42 |

^a^Pt layers are evaluated by (Diameter_Pd10@PtX_ - Diameter_Pd10_)/(2 × Pt (111) lattice spacing).

**Supplementary Table 3.** The Pt L_3_-edge EXAFS fitting parameters for Pd_10_@Pt_1_/UiO-66-NH_2_.

| Shell | R (Å)^a^ | N^b^ | σ^2^ (Å^2^)^c^ | R factor^d^ |
| --- | --- | --- | --- | --- |
| Pt-O | 2.009±0.073 | 0.8±0.4 | 0.0020±0.0015 | 0.006 |
| Pt-Pd | 2.789±0.021 | 6.1±2.7 | 0.0116±0.0042 |
| Pt-Zr | 2.673±0.034 | 3.6±3.0 | 0.0116±0.0042 |

^a^bond distance; ^b^coordination numbers; ^c^Debye-Waller factors; ^d^goodness of fit.

**Supplementary Table 4.** The Pt L_3_-edge EXAFS fitting parameters for Pd_10_@Pt_10_/UiO-66-NH_2_.

| Shell | R (Å)^a^ | N^b^ | σ^2^ (Å^2^)^c^ | R factor^d^ |
| --- | --- | --- | --- | --- |
| Pt-O | 2.034±0.020 | 0.6±0.5 | 0.0021±0.002 | 0.005 |
| Pt-Pd | 2.742±0.020 | 1.3±0.7 | 0.0094±0.002 |
| Pt-Pt | 2.750±0.036 | 8.1±2.3 | 0.0059±0.002 |

^a^bond distance; ^b^coordination numbers; ^c^Debye-Waller factors; ^d^goodness of fit.

**Supplementary Table 5.** The calculated Bader charge (eV) of Pt atom.

|  | SAA  (0.22 Pt layer) | Pt 1 layer | Pt 2 layers | Pt 3 layers |
| --- | --- | --- | --- | --- |
| Bader charge of each Pt atom | 0.206 | 0.089 | 0.014 | 0.008 |

**Supplementary Table 6.** The binding energy shift (eV) of Pt 4*f*_7/2_ based on XPS data.

|  | Pt layers^a^ | Binding energy shift |
| --- | --- | --- |
| Pd_10_@Pt_1_ | 0.31 | 0.55 |
| Pd_10_@Pt_10_ | 2.5 | 0.3 |

^a^Pt layers are evaluated by the Pd/Pt molar ratio.**Supplementary Table 7.** Calculated turn of frequency (TOF) of Pt for different catalysts.

| Cat | TOF_Pt_ |
| --- | --- |
| Pt_1_/UiO-66-NH_2_ | 13.4 |
| Pd_10_+Pt_1_/UiO-66-NH_2_ | 58.4 |
| Pd_10_Pt_1_/UiO-66-NH_2_ | 64.4 |
| Pd_10_@Pt_0.3_/UiO-66-NH_2_ | 213.6 |
| Pd_10_@Pt_1_/UiO-66-NH_2_ | 195.2 |
| Pd_10_@Pt_5_/UiO-66-NH_2_ | 54.3 |
| Pd_10_@Pt_10_/UiO-66-NH_2_ | 15.0 |

Apparently, Pd_10_@Pt_1_/UiO-66-NH_2_ is much more active than Pt_1_/UiO-66-NH_2_, Pd_10_+Pt_1_/UiO-66-NH_2_ and Pd_10_Pt_1_/UiO-66-NH_2_, indicating that the charge redistribution effect between Pd and Pt has a significant effect on the activity. In addition, the activity of Pd_10_@Pt_1_/UiO-66-NH_2_ is much higher than all other core-shell Pd@Pt/UiO-66-NH_2_, indicating that the regulation of charge redistribution effect also plays a significant role in the improvement of photocatalytic activity.

**References**

1. Hu Z, Peng Y and Kang Z *et al.* A modulated hydrothermal (MHT) approach for the facile synthesis of UiO-66-type MOFs. *Inorg Chem* 2015; **54**: 4862-8.
2. Schultz DM and Yoon TP. Solar synthesis: prospects in visible light photocatalysis. Science 2014; 343: 1239176.
3. Marcinkowski MD, Darby MT and Liu J et al. Pt/Cu single-atom alloys as coke-resistant catalysts for efficient C-H activation. Nat Chem 2018; 10: 325-32.
4. Duchesne PN, Li  ZY and Deming CP et al.  Golden single-atomic-site platinum electrocatalysts. Nat Mater 2018; 17: 1033-9.
5. Chen L, Huang B and Qiu X *et al.* Seed-mediated growth of MOF-encapsulated Pd@Ag core-shell nanoparticles: toward advanced room temperature nanocatalysts. *Chem Sci* 2016; **7**: 228-33.
6. Duchesne PN, Li  ZY and Deming CP *et al.*  Golden single-atomic-site platinum electrocatalysts. *Nat Mater* 2018; **17**: 1033-9.
7. Xiao JD, Shang Q and Xiong Y *et al.* Boosting photocatalytic hydrogen production of a metal-organic framework decorated with platinum nanoparticles: the platinum location matters. *Angew Chem Int Ed* 2016; **55**: 9389-93.
